# Sinus Lift with Collagenated Porcine Xenograft in Severely Atrophic Posterior Maxillae: Case Series with Histologic Correlation and Long-Term Outcomes

**DOI:** 10.3390/dj13120584

**Published:** 2025-12-05

**Authors:** Alexandru Spînu, Felicia Manole, Alexandru Burcea, Cristina-Crenguţa Albu, Lavinia-Florica Mărcuț, Roxana Daniela Brata, Alexia Manole, Claudia Florina Bogdan-Andreescu

**Affiliations:** 1 Doctoral School, Faculty of Medicine and Pharmacy, University of Oradea, 410068 Oradea, Romania; spinu.alexandru@didactic.uoradea.ro; 2 Spinu Dental Clinic, Spinu Learning, 410155 Oradea, Romania; 3 Surgical Disciplines Department, Faculty of Medicine and Pharmacy, University of Oradea, 410068 Oradea, Romania; fmanole@uoradea.ro (F.M.); lmarcut@uoradea.ro (L.-F.M.); 4 Department of Speciality Disciplines, “Titu Maiorescu” University, 031593 Bucharest, Romania; claudia.andreescu@prof.utm.ro; 5 Department of Genetics, Faculty of Dentistry, “Carol Davila” University of Medicine and Pharmacy, 020021 Bucharest, Romania; 6 Medical Disciplines Department, Faculty of Medicine and Pharmacy, University of Oradea, 410068 Oradea, Romania; brata.roxanadaniela@didactic.uoradea.ro; 7 Faculty of Medicine and Pharmacy, University of Oradea, 410068 Oradea, Romania; manole.alexia@student.uoradea.ro

**Keywords:** atrophic maxilla, bone regeneration, collagenated xenograft, dental implants, histomorphometry, osseointegration, porcine bone substitute, posterior maxilla, Schneiderian membrane, sinus floor elevation

## Abstract

**Background:** Maxillary sinus floor augmentation is widely used to enable implant placement in the atrophic posterior maxilla, yet comparative data for porcine-derived xenografts remain limited. **Objective:** To evaluate long-term bone regeneration and implant outcomes following sinus augmentation using a collagenated porcine xenograft. **Methods:** This paper reports a retrospective case series of three partially edentulous patients (aged 46–56 years) who underwent lateral sinus augmentation with a small-particle collagenated porcine xenograft (THE Graft™, Purgo Biologics, Gyeonggi-do, Republic of Korea) and staged implant placement. In one case, a controlled perforation of the Schneiderian membrane was performed to access and remove a sinus mucocele, followed by repair using a resorbable collagen membrane. Core biopsies were harvested at implant placement for histology (hematoxylin-eosin, Masson–Goldner) and tartrate-resistant acid phosphatase (TRAP) staining. Clinical outcomes included surgical events, vertical bone gain, marginal bone levels, and implant survival at long-term follow-up. **Results:** Healing was uneventful in all cases. Mean vertical bone gain was 12.0 mm (baseline 1.33 mm to 13.33 mm final). At a mean 46.8-month follow-up (range 38.3–52.2 months), 100% of implants were functional without failure; marginal bone loss remained < 1 mm during the first year and was stable thereafter. Histology at 3.7, 4.7, and 7.5 months showed vascularized new trabecular bone intimately contacting residual xenograft particles (new bone 20–30%, residual biomaterial 30–40%, connective tissue 30–50%). TRAP-positive multinucleated giant cells at 7.5 months indicated ongoing biomaterial degradation without severe inflammatory reactions. **Conclusions:** Within the limits of a small case series, collagenated porcine xenograft supported predictable bone regeneration and stable long-term implant function after sinus floor elevation, with favorable histologic integration and gradual resorption.

## 1. Introduction

Restoring edentulous areas in the posterior maxilla with dental implants can be challenging due to the frequent lack of adequate bone height. This deficiency appears from progressive alveolar ridge resorption after tooth extraction, combined with maxillary sinus pneumatization over time [1]. In addition to reduced bone volume, the posterior maxilla often presents low-density trabecular bone, which further compromises primary implant stability and long-term functional success [2,3].

First proposed in the late 1970s by Tatum and further developed by Boyne and James, maxillary sinus floor augmentation is today a safe, predictable, and well-established surgical procedure that increases vertical bone height in the atrophic posterior maxilla [4,5,6,7,8]. The technique has enabled clinicians to overcome the anatomic limitations of the posterior maxilla and achieve very high implant survival rates, making it one of the cornerstones in implant site development [9].

The primary objective of sinus floor augmentation is to obtain adequate bone volume to allow proper implant placement. There are two approaches-one-stage and two-stage-for accomplishing this goal [10]. In the one-stage procedure, the sinus is augmented, and the implant is placed during the same surgery, thereby reducing the total treatment time [11]. In the two-stage protocol, implant placement is delayed until the graft has healed and sufficient bone has formed. Although this staged approach typically requires a healing period of approximately six months before implant placement, it offers the advantage of enhanced control over graft integration and sinus healing, particularly in severely resorbed ridges [12].

The selection of grafting material strongly influences the long-term success of this procedure. For predictable bone regeneration, graft materials must demonstrate osteoconductive properties, promote neovascularization, and maintain dimensional stability during maturation [13].

A variety of graft materials exist, including autografts, allografts, xenogeneic materials, and synthetic biomaterials; yet, despite years of research, there is still no definitive gold standard [14] (Table 1). Autogenous bone is often regarded as the reference material, given its presence of osteogenic, osteoinductive, and osteoconductive properties; however, it is associated with increased surgical morbidity and limited availability [15,16]. Consequently, xenografts have become an attractive alternative, with porcine-derived xenografts gaining attention because of their biocompatibility, structural similarity to human bone, and potential for predictable bone regeneration [17,18].

The choice of graft form also influences clinical outcomes. Particulate grafts are widely used because they are easy to handle and adapt to small or contained defects, whereas block grafts are preferred in cases requiring significant horizontal or vertical reconstruction and long-term space maintenance [19]. In addition, xenografts can be combined with autogenous bone to enhance regenerative capacity by providing viable osteogenic cells and biologically active growth factors.

**Table 1:** Summative table with available graft materials for dental implant surgery.

Graft Material	Advantages	Disadvantages	Typical Indications	Precautions	References
Autogenous Bone (patient’s own bone—intraoral or extraoral donor site)	- Gold standard: osteogenic, osteoinductive, and osteoconductive - Excellent integration and remodeling - No risk of immune reaction or disease transmission	- Limited availability - Donor site morbidity (pain, infection, nerve injury) - Higher surgical time and complexity - Higher resorption rate over time	- Severe bone deficiency - Cases requiring maximum regenerative potential - Young, healthy patients willing to undergo additional surgery	- Careful donor site selection - Monitor for complications at the harvest site - Consider resorption rate when planning implant timing	[20,21,22]
Allografts (bone from human donor, processed and sterilized)	- Readily available - Avoids second surgical site - Good osteoconductivity, some osteoinductivity	- Possible risk of disease transmission (very low with proper processing) - Lower osteogenic potential compared to autografts	- Moderate bone defects - Patients who refuse or cannot undergo autogenous bone harvesting	- Use from reputable tissue banks - Inform the patient about the theoretical disease transmission risk	[20,23,24,25]
Xenografts (bone from animal source, e.g., bovine, porcine, equine)	- Unlimited supply - Excellent osteoconductivity - Slow resorption helps maintain volume long term - No donor site morbidity	- Lack of osteogenic cells - Slower remodeling compared to autografts - Possible patient concerns about animal origin or religious restrictions	- Sinus augmentation procedures where volume stability is critical - Large defects requiring space maintenance	- Ensure proper processing to avoid antigenicity - Obtain informed consent (religious/cultural considerations) - Monitor for delayed resorption in follow-up	[20,22,26]
Synthetic Alloplasts (calcium phosphate, HA, β-TCP, bioactive glass, etc.)	- Unlimited supply - No disease transmission risk - Customizable resorption rates - No donor site morbidity	- Only osteoconductive - May have less predictable long-term remodeling compared to autograft/xenograft - Some materials may fragment or resorb too quickly	- Small to moderate defects - Patients avoiding biologic materials (autograft/xenograft)	- Choose material with appropriate resorption profile for clinical need - Combine with PRF or growth factors to enhance results	[14,20,22,27]

A systematic review by Troeltzsch et al. [28] reported a mean horizontal gain of 3.7 ± 1.2 mm across all particulate grafting materials, with the lowest gain observed for synthetic substitutes (2.2 ± 1.2 mm) and the highest gain achieved by mixtures of autogenous bone with allogeneic or xenogeneic grafts (4.5 ± 1.0 mm) [28]. The weighted overall mean vertical gain was 3.7 ± 1.4 mm, with substantially greater vertical gain observed when space-maintaining barrier materials, such as titanium meshes, were used. Block grafts were shown to achieve approximately 1 mm more horizontal gain than particulate grafts. They produced significantly higher vertical augmentation only when autogenous block grafts harvested from extraoral donor sites were employed.

Nevertheless, the biological behavior of porcine-derived xenografts in sinus augmentation remains insufficiently characterized. In particular, data on their ability to support histological new bone formation and graft integration are still scarce compared to other xenografts.

This study investigates new bone formation and graft integration following maxillary sinus floor augmentation using a collagenated porcine-derived xenograft in three patients. Clinical parameters include surgical success, implant stability, and complication rates, while histologic analysis focuses on the proportion of newly formed bone, residual graft particles, and the amount of connective tissue. Therefore, this study evaluates the clinical, radiographic, and histological outcomes of maxillary sinus floor augmentation using a porcine-derived xenograft in a case series of three patients. Particular attention was given to the amount of new bone, the resorption of xenograft material, as well as the performance of implants at long-term follow-up.

Human histological evaluation after sinus augmentation is challenging, and animal studies or radiographic assessments prevail in the current literature without tissue analysis. This case series aims to contribute to an underrepresented area of research by providing detailed histological and cone-beam computed tomography (CBCT)-correlated data from well-documented clinical cases. These findings provide meaningful evidence for understanding graft remodeling and bone regeneration in the posterior maxilla.

## 2. Materials and Methods

### 2.1. Study Design and Ethical Approval

This is a retrospective case series of three patients who required implant-supported rehabilitation in the posterior maxilla due to insufficient residual bone height (RBH). All clinical procedures were performed between May 2021 and November 2021 as part of routine dental care. The study was conducted in accordance with the Declaration of Helsinki and received ethical approval for the retrospective analysis and publication of anonymized data from the Bioethics Committee of Dr. Spinu Dental Clinic, Oradea (Approval No. 01/25.11.2024). All patients provided written informed consent for treatment and the use of de-identified clinical/radiologic data, as well as histology, for research purposes.

### 2.2. Patient Selection

Between 3 May and 30 November 2021, 83 patients underwent sinus augmentation procedures at our clinic. Eligibility criteria for inclusion in this retrospective analysis were: severely atrophic posterior maxilla with residual bone height ≤ 2 mm requiring maxillary sinus lift; absence of active sinus pathology; lateral window sinus floor elevation performed using collagenated porcine bone mineral; return for implant placement during the study period, allowing clinically indicated bone biopsy; non-smokers status; excellent general health status (score I according to American Society of Anesthesiologists classification [29]); complete clinical and radiographic documentation; and written informed consent for the scientific use of anonymized clinical, radiographic, photographic, and histological data.

Exclusion criteria included smoking, systemic conditions affecting bone healing, incomplete records, different grafting materials, or refusal of consent. Three patients fulfilled all criteria and were included in the study.

**Figure 1:**
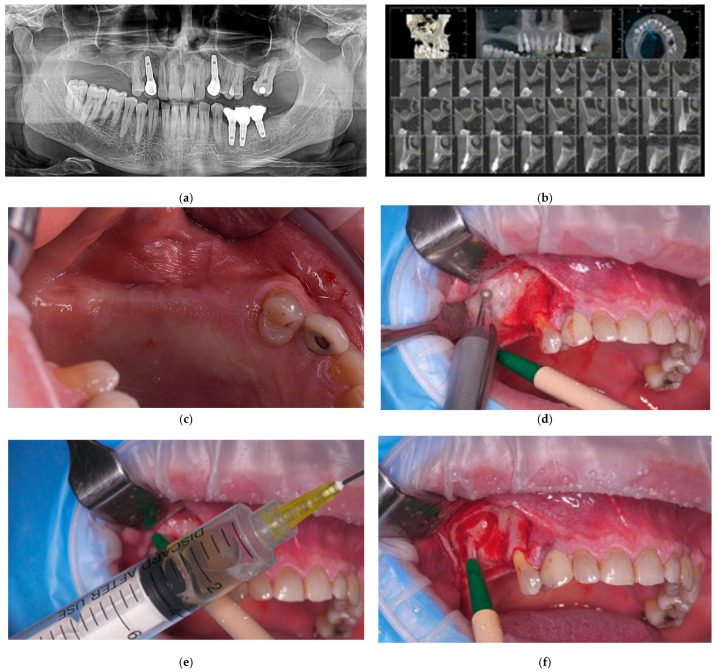

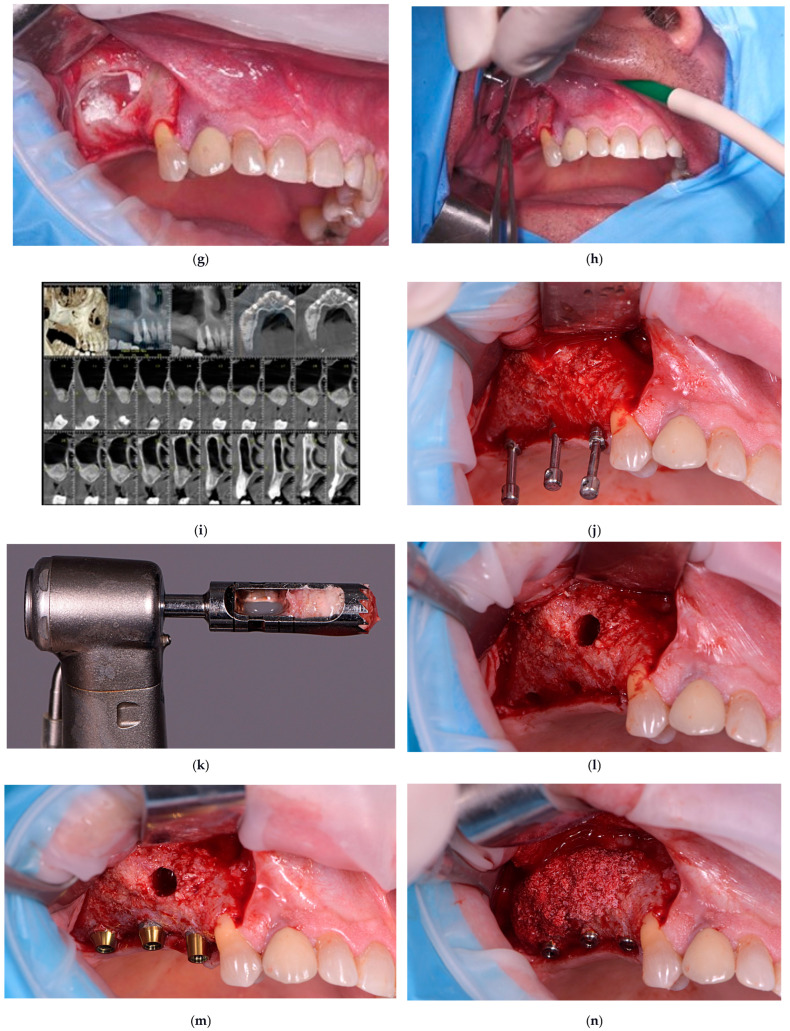

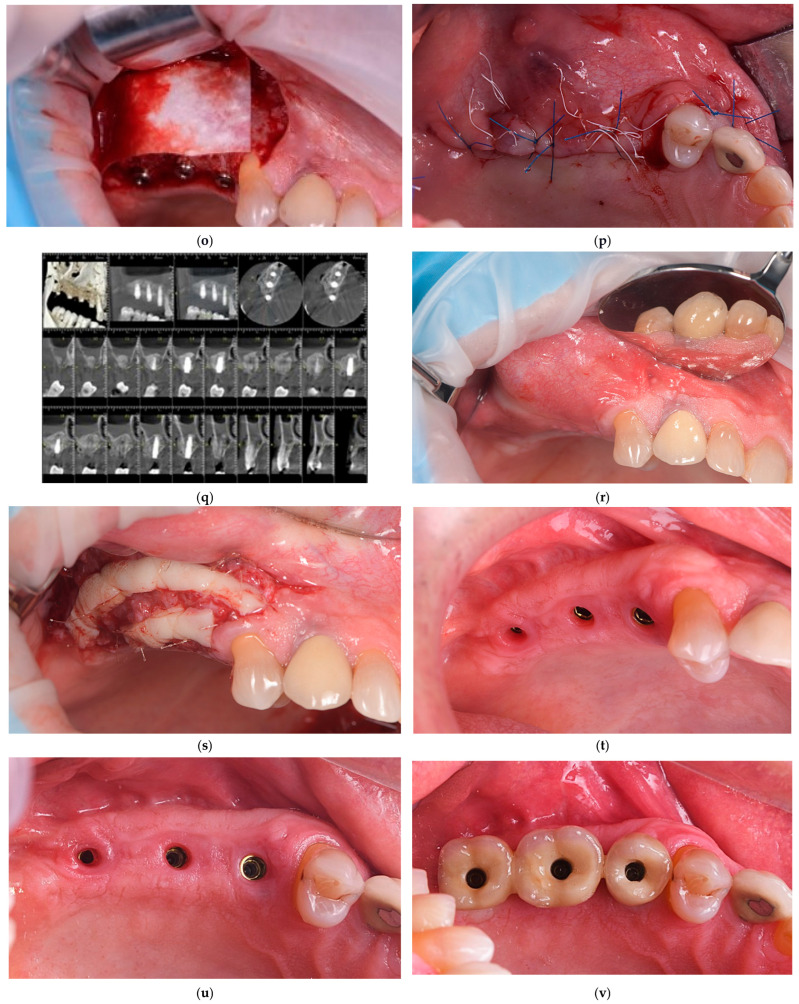

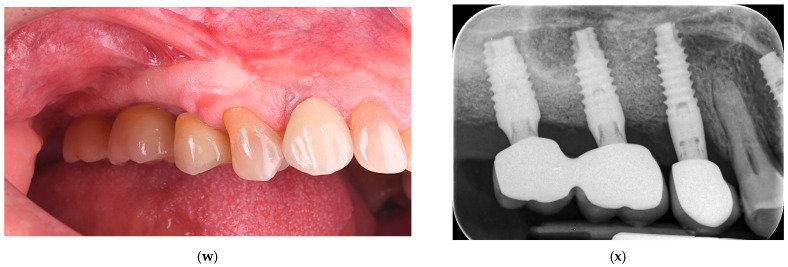
Representative clinical and radiographic images of sinus floor augmentation and implant rehabilitation. (**a**) OPG showing a previous implant-supported crowns and a edentulous span in the right posterior maxilla with significant bone loss, unfavorable for implant placement; (**b**) Preoperative CBCT scan confirming insufficient residual bone and revealing the presence of a maxillary mucocele; (**c**) Preoperative aspect of posterior atrophic maxilla; (**d**) Lateral bony window preparation with a round diamond bur under copious irrigation; (**e**) Aspiration of mucocele content prior to enucleation; (**f**) Surgical enucleation of the maxillary mucocele; (**g**) Repairing of the Schneiderian membrane perforation with a collagen membrane, followed by filling of the subantral cavity with small-particle (0.25–1.00 mm) collagenated porcine-derived xenograft (THE Graft™, Purgo Biologics Inc., Gyeonggi-do, Republic of Korea); (**h**) Coverage of the grafted sinus cavity with a resorbable collagen membrane prior to flap repositioning and suturing; (**i**) Postoperative CBCT after lateral sinus lift; (**j**) Intraoperative view of the buccal bone at 3.7 months after lateral sinus lift. Osteotomies are prepared for implant placement during crestal sinus lift with simultaneous implantation; (**k**) Bone biopsy specimen harvested from the grafted maxillary sinus using a trephine bur at the time of implant placement; (**l**) Bone biopsy site and prepared osteotomies for implant placement; (**m**) Prama^®^ (Sweden & Martina, Due Carrare, Italy) dental implants placed to replace missing teeth 1.5., 1.6., and 1.7. following crestal sinus floor augmentation; (**n**) The biopsy site filled with small-particle (0.25–1.00 mm) collagenated porcine-derived xenograft (THE Graft™, Purgo Biologics Inc., Gyeonggi-do, Republic of Korea); (**o**) Xenograft cover with resorbable collagen membrane; (**p**) Flap closure and suturing, (**q**) Postoperative CBCT scan showing augmented sinus and dental implants in place, demonstrating adequate bone volume and implant stability, (**r**) Clinical view of the posterior maxilla three months after crestal sinus lift, showing insufficient keratinized mucosa; (**s**) Vestibuloplasty with apically repositioned flap and palatal connective tissue graft in the posterior maxilla, performed to improve peri-implant soft tissue conditions; (**t**) Three-month postoperative appearance of the buccal vestibule following vestibuloplasty, with healthy keratinized mucosa; (**u**) Clinical intraoral view of uncovered dental implants and healthy peri-implant soft tissues.; (**v**) Three screw-retained porcelain-fused-to-metal implant-supported crowns in place; (**w**) Implant-supported crowns with peri-implant soft tissues healthy and well adapted; (**x**) Periapical radiograph of the posterior maxilla after implant loading, showing good osseointegration, stable crestal bone levels, and healthy peri-implant conditions.

Three partially edentulous patients (two males and one female; age range: 46–56 years) were enrolled. All patients sought implant-supported rehabilitation to restore esthetics and masticatory function in the posterior maxilla. An orthopantomogram (OPG) (Figure 1a) and cone-beam computed tomography (CBCT) (Figure 1b) scan were performed for each patient to thoroughly evaluate the surgical site and determine the optimal treatment approach.

Based on the CBCT findings and a detailed discussion with each patient, the treatment plan for severely atrophic maxilla with residual bone height ≤ 2 mm (Figure 1c) consisted of lateral sinus floor elevation with staged implant placement, followed by prosthetic restoration six months after implant placement.

This case series included all consecutive patients who underwent lateral sinus augmentation with the same collagenated porcine xenograft and staged implant placement during the study period, and in whom core biopsies could be obtained ethically. All specimens were processed using the same histologic protocol to ensure consistent evaluation of bone formation and graft remodeling.

### 2.3. Surgical Procedure

#### 2.3.1. Preoperative Preparation

All patients received antibiotic prophylaxis with amoxicillin and clavulanic acid 875/125 mg (Augmentin^®^, GlaxoSmithKline, London, UK), prescribed at a dosage of one tablet every 12 h, starting 24 h before surgery and continued for seven days. Antibiotic prophylaxis is part of the standard clinical protocol for lateral sinus augmentation with grafting at Spînu Dental Clinic. Although recent systematic reviews report limited evidence supporting routine prophylactic antibiotic use for sinus elevation procedures [30,31], our protocol values infection prevention due to the potential severity of postoperative sinusitis or graft infection in procedures that involve communication with the maxillary sinus. None of the patients in this series were immunocompromised or presented systemic conditions requiring antibiotic therapy. The decision to administer perioperative antibiotics was based on the surgeon’s judgment and institutional clinical protocol to reduce the risk of postoperative complications in this high-risk anatomical region.

Immediately before anesthesia, patients performed an oral rinse with 0.2% chlorhexidine solution to reduce the bacterial load in the oral cavity. Local anesthesia was achieved using articaine hydrochloride 4% with epinephrine 1:100,000 (Ubistesin forte^®^, 3M ESPE, Seefeld, Germany), administered via posterior superior alveolar and greater palatine nerve blocks.

#### 2.3.2. Surgical Steps

A crestal incision with vertical releasing incisions was made to provide adequate access, and a full-thickness mucoperiosteal flap was elevated. In two patients, non-restorable teeth within the surgical field were extracted atraumatically to preserve the alveolar bone. A lateral window, approximately 8 × 6 mm in size, was prepared using a 3 mm round diamond bur (Figure 1d).

The Schneiderian membrane was carefully elevated without perforation. In one case, an asymptomatic mucocele was enucleated during the sinus lift using the previously described Crock-eye technique [32] (Figure 1e,f). This required intentional perforation of the Schneiderian membrane, which was subsequently repaired with a BioCover™ membrane (Figure 1g). The subantral cavity was then grafted with small-particle (0.25–1.00 mm) collagenated porcine-derived xenograft material (approximately 2 g; THE Graft™, Purgo Biologics Inc., Gyeonggi-do, Republic of Korea) mixed with autologous blood (Figure 1g). The grafted site was covered with a 30 × 40 mm resorbable collagen membrane (BioCover™, Purgo Biologics Inc., Gyeonggi-do, Republic of Korea) (Figure 1h). Flaps were repositioned and closed with tension-free primary closure using 4-0 PTFE sutures (Biotex™, Purgo Biologics Inc., Gyeonggi-do, Republic of Korea), and an immediate postoperative CBCT scan was performed (Figure 1i).

A delayed implant placement protocol was chosen due to the reduced residual cortical bone height. Dental implants used in this study included Prama^®^ (Sweden & Martina, Due Carrare, Italy) and Straumann^®^ implants (Straumann Group, Basel, Switzerland), selected according to prosthetic requirements and clinical indications.

Because of the presence of the maxillary mucocele in one case, there was insufficient bone volume for the placement of standard implants (12–13 mm length, 4 mm diameter). Therefore, during the second-stage procedure, an internal (crestal) sinus lift was additionally performed in combination with implant placement (Figure 1j).

At the time of implant placement, bone biopsies were harvested from the grafted sites using a 5 mm trephine bur (Figure 1k,l). Following implant placement (Figure 1m), the biopsy sites were re-grafted with small-particle (0.25–1.00 mm) collagenated porcine-derived xenograft (THE Graft™, Purgo Biologics Inc., Gyeonggi-do, Republic of Korea) (Figure 1n), covered with a resorbable collagen membrane (BioCover™, Purgo Biologics Inc., Gyeonggi-do, Republic of Korea) (Figure 1o), and the surgical wound was closed with sutures (Figure 1p). A postoperative CBCT scan was obtained to confirm the position of the implants and the graft (Figure 1r).

Three months after implant placement, clinical evaluation revealed insufficient keratinized gingiva for successful prosthetic reconstruction (Figure 1q). Therefore, a palatal connective tissue graft was performed to enhance peri-implant mucosal conditions (Figure 1s).

#### 2.3.3. Postoperative Care

Postoperative management included administration of nimesulide 100 mg (Aulin^®^, Angelini Pharma, București, Romania) every 12 h to control pain and inflammation. Patients were advised to apply cold packs for the first 48 h, maintain a soft diet until the sutures are removed, and sleep with their head elevated to minimize swelling. Oral hygiene was maintained with saline rinses while avoiding brushing at the surgical site. Strenuous activity, nose blowing, and closed-mouth sneezing were restricted for 2–4 weeks to protect graft stability. Mild discomfort, swelling, and minor nasal bleeding were considered normal; patients were instructed to report persistent bleeding or unusual symptoms immediately.

### 2.4. Biopsy Collection and Processing

Core biopsies were not part of a routine protocol for sinus augmentation at our institution. In the present three cases, biopsy collection was clinically indicated at the time of implant osteotomy due to the severely atrophic posterior maxilla and the need to verify bone quality before placement of standard implants. Patients received detailed information and provided written consent for both the biopsy procedure and the scientific use of anonymized tissue samples. No biopsy was taken solely for research purposes, and no additional surgery or morbidity was introduced.

Biopsies harvested at implant placement were obtained from the buccal wall adjacent to the osteotomy, containing newly formed bone and xenograft material without cortical bone.

Specimens were immediately fixed in 10% buffered formalin for 48 h. Decalcification was performed in 10% tris-buffered ethylenediamine tetraacetic acid (EDTA; Carl Roth, Karlsruhe, Germany) at 37 °C for 7 days using an ultrasonic decalcifier (Medite, Dietikon, Switzerland). Following decalcification, samples were dehydrated through a graded alcohol series and cleared in xylol.

The processed specimens were embedded longitudinally in paraffin, and 2–4 μm sections were obtained from the central region using a microtome (Leica, Wetzlar, Germany). Sections were stained with hematoxylin and eosin (HE), Masson–Goldner trichrome, and tartrate-resistant acid phosphatase (TRAP) according to standard protocols.

### 2.5. Histological Evaluation

Histomorphometric evaluation was performed according to a standardized study protocol using a research-grade scanning microscope combined with NIS-Elements software (Nikon, Tokyo, Japan). Digital images of the entire implantation beds (“total scans”), including the bone substitute material and surrounding peri-implant sinus tissue, were acquired with a DS-Fi1 digital camera (Nikon, Tokyo, Japan) connected to an Eclipse 80i microscope (Nikon, Tokyo, Japan) equipped with an automated scanning stage (Prior, Rockland, MA, USA).

The resulting image datasets were obtained at a magnification of ×200. For illustrative purposes, representative higher-magnification images of relevant structures were extracted from the total scans using Photoshop software (Adobe, San Jose, CA, USA).

Histological slides were evaluated by an experienced oral pathologist who was not involved in the surgical procedures. Although formal coding and blinding procedures were not implemented, the evaluator was not informed of the clinical outcomes during microscopic assessment, in order to minimize interpretation bias.

### 2.6. Postoperative and Follow-Up Protocol

Patients were monitored through scheduled follow-ups at 24 h, one week, one month, three months, and six months postoperatively. A CBCT was obtained immediately after surgery to confirm the positioning of the graft. At the one-week follow-up, clinical evaluation included assessment of pain, soft tissue healing, swelling, infection, and suture integrity. Patients reported only mild discomfort, which was effectively managed with prescribed analgesics. No signs of infection, wound dehiscence, or excessive swelling were observed.

At the one- and three-month follow-ups, soft tissue adaptation and the absence of sinus-related complications (nasal congestion, sinusitis) were confirmed. Healing was uneventful in all cases, with no pain or inflammation. Standard-length implants (12–13 mm length, 4 mm diameter) were placed after a healing period of 4–8 months.

Six months after implant placement, the posterior maxilla showed adequate bone volume and sufficient fixed keratinized gingiva (Figure 1t). Porcelain fused-to-metal screw-retained implant-supported crowns were delivered (Figure 1u–w). All implants demonstrated excellent primary and secondary stability, with no mobility, discomfort, or inflammation. Radiographic evaluation confirmed stable marginal bone levels and the absence of peri-implant radiolucency (Figure 1x).

**Figure 2:**
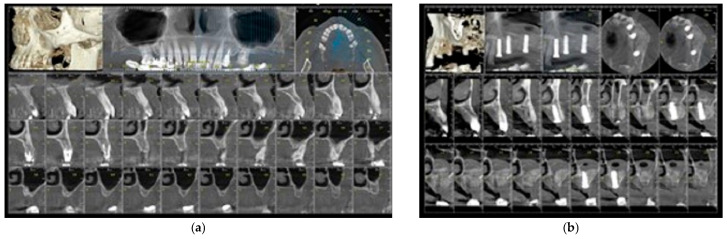

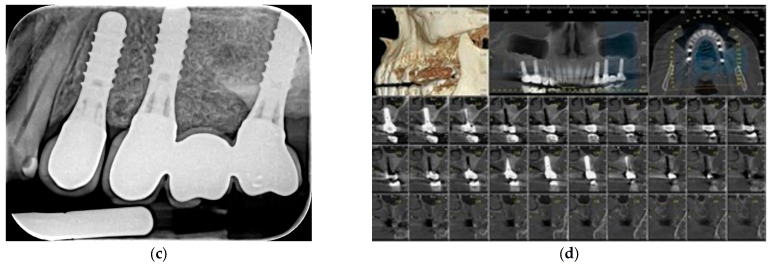
Representative radiographic follow-up. (**a**) Preoperative CBCT scan confirming severe maxillary atrophy; (**b**) Postoperative CBCT scan obtained immediately after placement of Straumann^®^ implants (Straumann Group, Basel, Switzerland), showing accurate implant positioning, restored maxillary sinus floor height, and stable graft integration; (**c**) Retro-alveolar periapical radiograph following prosthetic loading, proving stable crestal bone levels and intimate bone–implant contact are visible around implants; (**d**) Postoperative CBCT scan at 52.23 months demonstrating long-term stability of the augmented sinus floor and successful osseointegration.

Annual follow-ups were performed thereafter, focusing on implant success, peri-implant bone stability, and patient satisfaction. During long-term follow-up (mean 46.8 months), no implant failures were recorded. Marginal bone loss remained within acceptable limits, and no sinus-related complications were observed (Figure 2).

This protocol allowed a consistent evaluation of clinical healing, graft integration, and implant survival across all three cases. This case series was conducted and reported in accordance with the CARE (CAse REport) guidelines, with compliance summarized in Table 2.

## 3. Results

### 3.1. Clinical Outcomes

Three patients (two males and one female; mean age, 50.3 years; range, 46–56 years) underwent maxillary sinus augmentation using small-particle (0.25–1.00 mm) collagenated porcine-derived xenograft (THE Graft™, Purgo Biologics Inc., Gyeonggi-do, Republic of Korea) and staged implant placement (Table 3). In one case, a lateral sinus lift was followed by a crestal sinus lift with simultaneous implant placement due to limited residual bone after the initial augmentation.

No intraoperative or postoperative complications were recorded, except for one intentional Schneiderian membrane perforation during mucocele removal, which was successfully repaired with a collagen membrane. All sites healed uneventfully.

Radiographic analysis showed a mean vertical bone gain of 12.0 mm, increasing from a baseline of 1.33 mm to a final mean of 13.33 mm. Implants achieved good primary stability, and screw-retained prosthetic crowns were delivered six months after implant placement.

At long-term follow-up (mean 46.8 months, range: 38.3–52.2 months), all implants remained functional without failure, mobility, or peri-implant infection (Figure 2). No sinus-related complications were observed.

### 3.2. Histological and Histomorphometric Outcomes

Bone biopsies were obtained at 3.7, 4.7, and 7.5 months post-augmentation (Table 4). All specimens showed new trabecular bone (NB) formation, integration of xenogenic bone substitute material (BSM), and evidence of vascularization.

**Figure 3:**
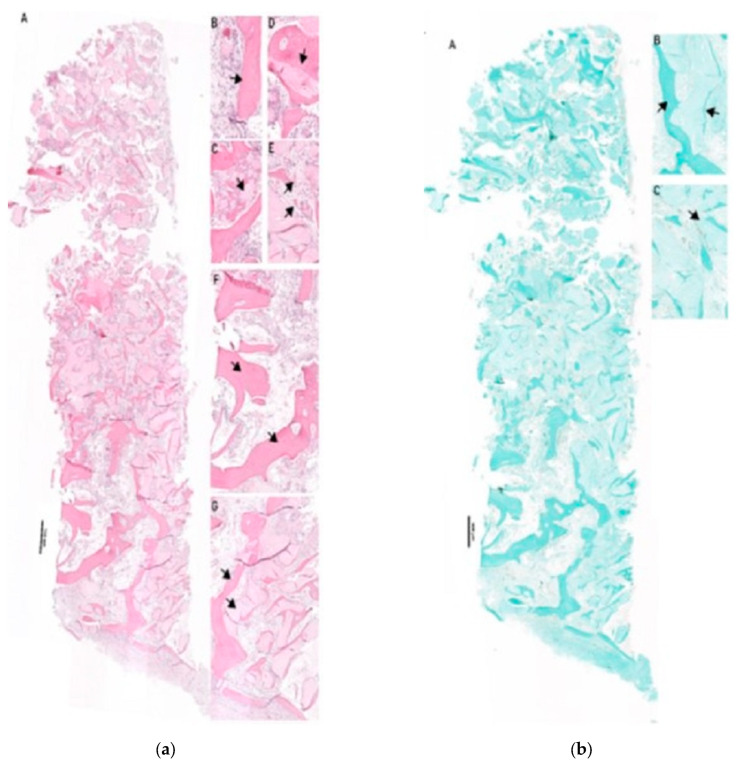
(**a**) Histology of biopsy C1221-3. (A): total scan of biopsy. (B–E): representative images of evaluated structures, arrow(s) indicate relevant areas and/or structures. (B): NB formation, (C): residual BSM, (D): hybrid bone formation, (E): vessel formation, (F): residual bone area, (G): residual bone and adjacent BSM with NB formation. Staining: H/E staining of paraffin histological sections. (**b**) Histology of biopsy C1221-3. (**b**) Histology of biopsy C1221-3 (Masson Goldner). (A): total scan of biopsy. (B,C): representative images of evaluated structures, arrow(s) indicate relevant areas and/or structures. (B): NB formation at autologous bone compared to xenogenic BSM, (C): hybrid bone formation. Staining: Masson Goldner staining of paraffin histological sections.

**Figure 4:**
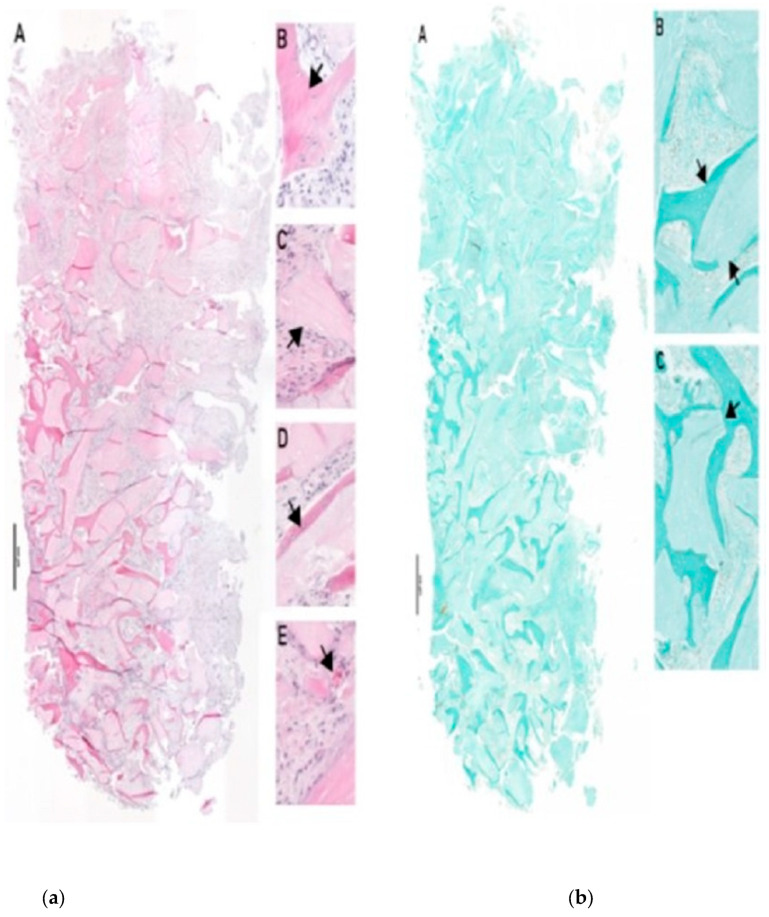
(**a**) Histology of biopsy C1221-1 (HE). (A): total scan of biopsy. (B–F): representative images of evaluated structures, arrow(s) indicate relevant areas and/or structures. (B): NB formation, (C): residual BSM, (D): hybrid bone formation, (E): vessel formation. Staining: H/E staining of paraffin histological sections. (**b**) Histology of biopsy C1221-1 (Masson Goldner). (A): total scan of biopsy. (B,C): representative images of evaluated structures, arrow(s) indicate relevant areas and/or structures. (B): NB formation at autologous bone compared to xenogenic BSM, (C): hybrid bone formation. Staining: Masson Goldner staining of paraffin histological sections.

**Figure 5:**
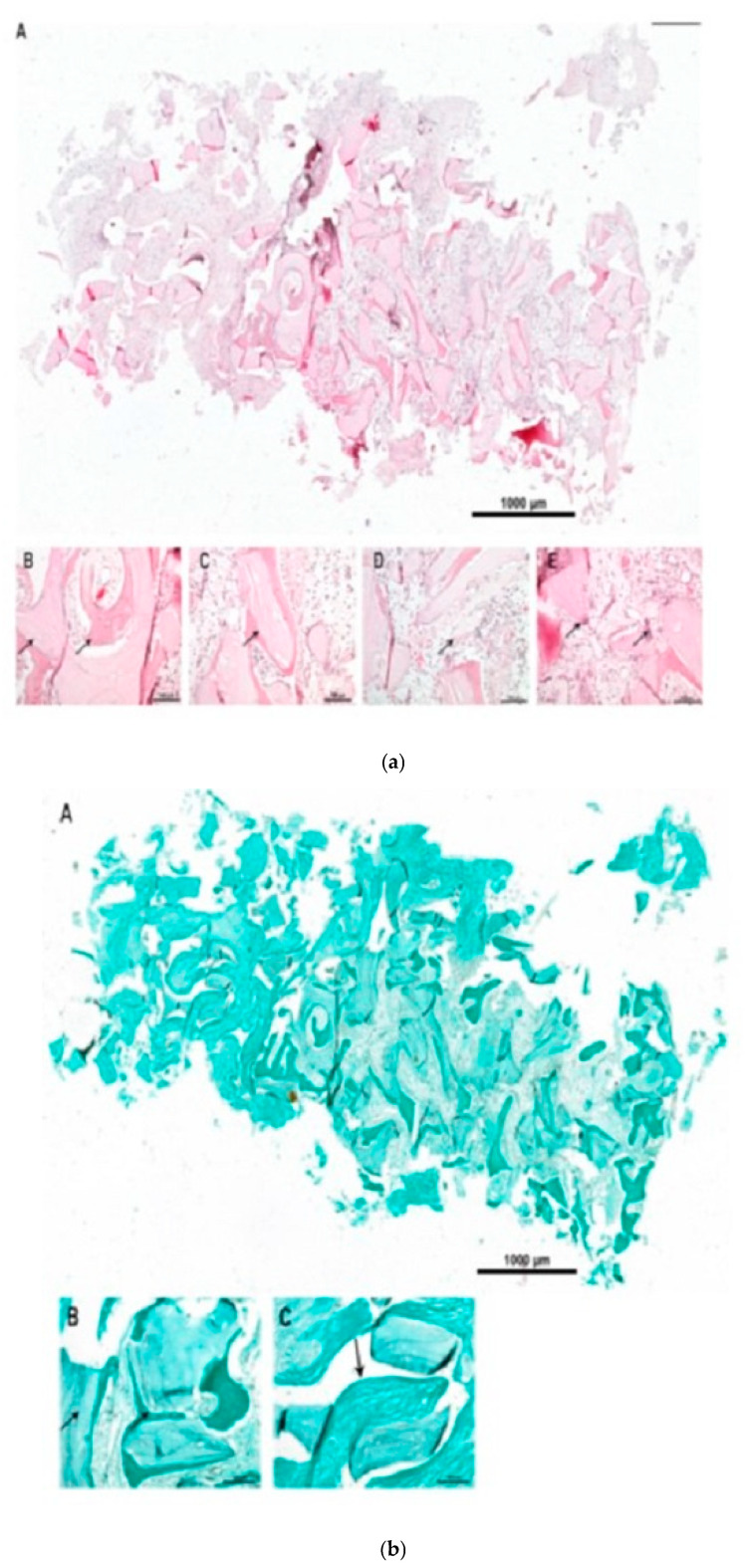

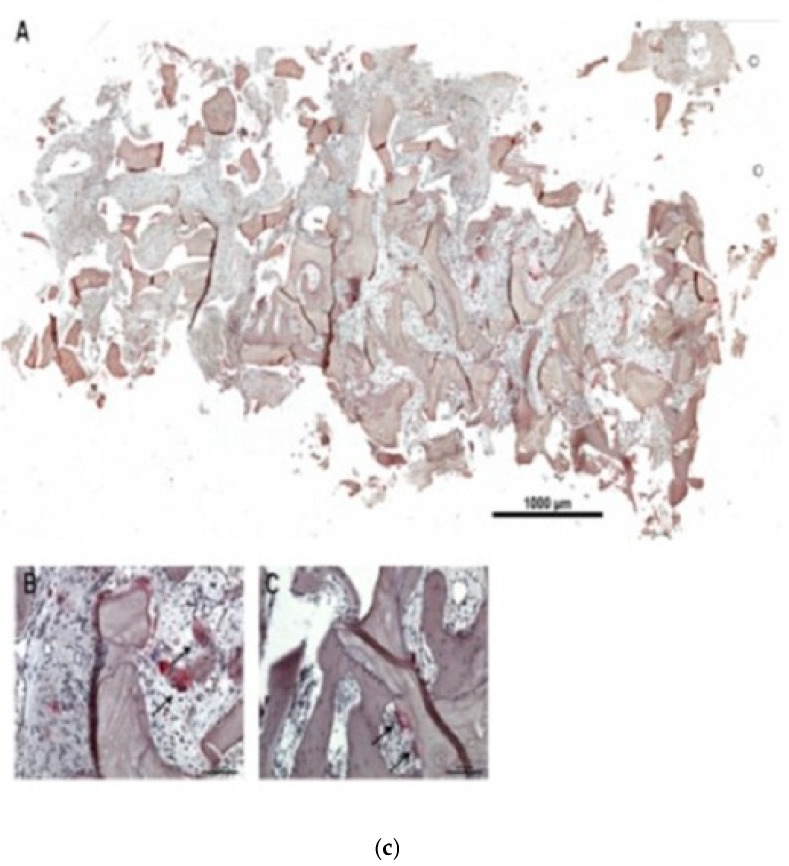
(**a**) Immunohistology of biopsy C0322-1 (HE). (A): total scan of biopsy. (B–E): representative images of evaluated structures, arrow(s) indicate relevant areas and/or structures. (B): residual BSM and NB formation, (C): hybrid bone formation, (D): vessel formation, and (E): MNGC. (**b**) Histology of biopsy C0322-1 (Masson Goldner). (A): total scan of biopsy. (B,C): representative images of evaluated structures, arrow(s) indicate relevant areas and/or structures. (B): mineralized bony structures and residual BSM, (C): BSM embedded into collagen rich CT (total scan, contra-lateral (left)). Staining: Masson Goldner staining of paraffin histological sections. (**c**) Immunohistology of biopsy C0322-1 (TRAP). (A): total scan of biopsy. (B,C): representative images of evaluated structures, arrow(s) indicate relevant areas and/or structures. (B,C): representative images for BSM surface with TRAP-positive cell accumulation, (C): TRAP-positive MNGC degrading residual BSM. Staining: Anti-TRAP (Tartrate-resistant acid phosphatase) immunohistological staining of paraffin histological sections.

- Bone biopsy no. C-1221-3, at 4.7 months after surgery, (Figure 3a,b): Residual bone was visible in the crestal and lateral regions. Approximately 30% NB, 30% connective tissue (CT), and 40% BSM were observed. Hybrid bone formation was evident with the incorporation of BSM granules into NB. Vascularization was well developed, with endothelial wall maturation visible. A mild foreign-body reaction was noted, without TRAP-positive multinucleated giant cells (MNGCs).
- Bone biopsy no. C-1221-1, at 3.7 months after surgery, (Figure 4a,b): No residual bone was detected. The biopsy contained ~20% NB, 50% CT, and 30% BSM. NB was distributed predominantly laterally, with hybrid bone formation and vascularization showing early endothelial maturation. No significant inflammation or TRAP-positive MNGCs were present, indicating a nearly complete degradation process.
- Bone biopsy no. C-0322-1 at 7.5 months after surgery, (Figure 5a–c): The biopsy revealed ~20% NB, 50% CT, and 30% BSM. NB was concentrated in the lateral portion of the specimen, while the contralateral part contained BSM embedded in collagen-rich CT without NB. Vascularization was present with vessels of varying sizes, some showing mature endothelial characteristics. TRAP-positive MNGCs were observed, suggesting ongoing BSM degradation.

Across all samples, no severe inflammation or adverse host reactions were observed.

**Table 3:** Clinical, surgical, and histological data of patients treated with sinus floor augmentation using porcine-derived xenograft.

No.	Gender	Age	Date of Surgery	Type of Surgery	Complications During Surgery	Major Complications After Surgery	Histology (Months)	Implant Site *	Bone Height (mm) Initial Final Gained			Follow-Up at 30 September 2025 (Months) **
1.	F	49	24 May 2021	Lateral sinus lift with staged implantation	none	none	4.7	2.5.; 2.6.; 2.7.	2	15	13	52.23
2.	M	56	9 August 2021 30 November 2021	Lateral sinus lift Crestal sinus lift with simultaneous implantation Connective tissue graft	Intentional perforation for mucocele removal	none	3.7	1.5.; 1.6.; 1.7.	1	10	9	49.73
3.	M	46	22 July 2021	Lateral sinus lift with staged implantation		none	7.5	2.6.; 2.7.	1	15	14	38.3
Mean value	-		-	-	-	-	5.3	-	1.33	13.33	12.0	46.75

* FDI tooth-numbering system, ** After sinus lift surgery.

**Table 4:** Histological evaluation of bone biopsies after sinus augmentation with porcine-derived xenograft.

No.	Time of Histology (Months)	Evaluation of Bone Regeneration and Biomaterial Integration	Evaluation of Immunological Response and Biomaterial Degradation	Overall Evaluation
1. C-1221-3	4.7	~30% NB, hybrid bone on BSM surfaces, well-integrated with CT; vascularization with maturing vessels	Mild foreign-body MNGCs, TRAP-negative	Ongoing regeneration; NB stimulated by BSM without adverse findings
2. C-1221-1	3.7	~20% NB, mainly lateral; hybrid bone with BSM integration; good vascularization.	No severe inflammation; no TRAP-positive MNGCs	Active regeneration; ~2/3 of sample with bony structures
3. C-0322-1	7.5	~20% NB, mainly lateral; BSM well integrated; contralateral area CT-rich with less NB	TRAP-positive MNGCs indicating active degradation	Ongoing degradation and regeneration; NB mainly lateral

BSM—bone substitute material; NB—new trabecular bone; CT—connective tissue, TRAP—tartrate-resistant acid phosphatase, MNGC—multinucleated giant cell.

These findings demonstrate that porcine-derived xenograft can achieve predictable bone regeneration in sinus floor augmentation, with stable long-term implant survival. The following discussion compares our results with previously published literature.

## 4. Discussion

This retrospective case series evaluated the clinical, radiographic, and histological outcomes of sinus floor augmentation using a small-particle (0.25–1.00 mm) collagenated porcine-derived xenograft (THE Graft™, Purgo Biologics Inc., Gyeonggi-do, Republic of Korea) in combination with staged implantation. The results demonstrated good healing, with significant vertical bone gain and 100% implant survival, accompanied by no major complications in the postoperative period. Histological examination confirmed the formation of NB, vascularization, and progressive xenograft resorption, testifying to good biocompatibility and osteoconductive properties of the material.

### 4.1. Clinical Healing and Vertical Bone Gain

Healing was uneventful in all patients, including one case that required controlled membrane perforation to access and remove a sinus mucocele. The Schneiderian membrane was repaired using a resorbable collagen membrane, and normal sinus healing was observed, corroborating reports that well-managed sinus membrane perforations do not compromise clinical outcomes and can be predictably repaired [11,33,34].

The present series included only three cases, which limits the ability to generalize the findings. At the same time, Schneiderian membrane perforation is one of the most frequently reported complications in the literature [35,36,37]. No intraoperative or postoperative complications occurred in our cohort, and all implants osseointegrated successfully.

It is important to note that one Schneiderian membrane perforation was performed intentionally to access and remove a sinus mucocele and was subsequently repaired without sequelae. No accidental perforations or other complications were observed. The absence of complications in this small case series reflects the limited sample size, careful patient selection, adherence to a standardized surgical protocol, and clinician experience with lateral window sinus augmentation.

The achieved vertical bone gain averaged 12 mm, consistent with, or surpassing, the results from sinus augmentation using bovine xenografts [38], allografts [39], or synthetic biomaterials [40]. A meta-analysis from 2011 [41] reported a maximum vertical gain of 4.62 mm, favoring grafting with either a mixture of autogenous bone and bovine-derived xenograft or no graft at all.

More recently, Orth et al. [42] confirmed a mean gain of 3.4 mm with simultaneous implantation when demineralized bovine bone mineral, hydroxyapatite+β-TCP, calcium phosphosilicate, or autologous leukoplatelet fibrin were used as grafting materials [42]. Vertical bone gain is significantly higher with the lateral approach compared to the crestal approach [43].

Xenografts offer good space-maintaining ability because of the slow resorption; the findings of the present investigation also support the predictable dimensional stability of xenografts.

Xenografts were found to exhibit the least resorption among graft materials in the early healing phase, while autogenous bone showed the greatest reduction in volume [44]. Additionally, xenograft in combination with autogenous bone or allograft demonstrated the highest bone gain compared to other graft materials [45]. Bovine-derived xenografts remain clinically and radiologically stable after a five-year follow-up [46]. In addition, xenografts, and particularly porcine-derived xenografts, are easy to handle and can be stabilized during surgery [26,47], making them suitable for use in major sinus lift surgeries, such as those with ≤2 mm of residual bone height.

Emerging evidence highlights the excellent physicochemical properties of porcine xenografts, which are comparable to those of bovine substitutes [17,48,49]. Proteomic studies have shown similarity between porcine graft matrices and clinically accepted human demineralized bone products [50]. Greater porosity and interconnected pore systems in porcine xenografts [48,51] may facilitate vascular infiltration and osteogenesis [52], supporting the enhanced angiogenic activity observed in this series. At the same time, slightly faster resorption of porcine xenografts compared to bovine has been reported [26,52], which may result in reduced long-term volume but increased new bone turnover—an advantageous characteristic when early bone maturation is desired.

Although many studies have been devoted to bovine grafts, the porcine-derived grafts have evolved as a suitable alternative and provide similar or even superior outcomes. However, studies concerning porcine grafts are still developing compared to bovine options.

### 4.2. Histological Findings and Bone Formation Dynamics

Histological evaluation provided evidence of NB formation and material integration across different healing intervals (3.7–7.5 months). All samples demonstrated hybrid bone formation, characterized by the incorporation of collagenated porcine-derived xenograft granules, into newly formed trabeculae. This phenomenon, previously described as a hallmark of stable biomaterial integration [26,53], highlights the capacity of the xenograft to act as a long-term osteoconductive scaffold.

Quantitative analysis revealed relative proportions of NB (20–30%), residual biomaterial (30–40%), and CT (30–50%), which are comparable to histomorphometric outcomes reported for collagenated bovine-derived xenografts [54,55]. These findings suggest that porcine xenografts possess similar osteoconductive potential to their bovine counterparts.

New vascularization was evident in all specimens. In later biopsies, endothelial wall maturation was observed, underlining the collagenated porcine-derived xenograft’s (THE Graft™, Purgo Biologics Inc., Gyeonggi-do, Republic of Korea) ability to support angiogenesis, a critical prerequisite for graft remodeling and long-term implant success.

### 4.3. Biomaterial Resorption and Host Response

Histological investigations have demonstrated favorable healing patterns after sinus lift augmentation. No pathologic lymphocyte infiltration or severe inflammatory reactions were found at 7.5 months. In general, only a mild foreign-body response was elicited, characterized by small numbers of MNGCs. TRAP staining demonstrated progressive biomaterial resorption, indicating further degradation during the period under investigation.

After the sinus floor elevation procedure, it is expected that the initial height of the grafted bone will resorb over time; however, this resorption generally slows down and stabilizes after the first 6–12 months. This pattern is closely related to the residual bone height at the time of surgery [56]. A 2014 study reported no significant differences in the resorption pattern between the lateral and crestal approaches or among different grafting combinations, including allograft mixed with autograft, bovine-derived xenograft mixed with autograft, or allograft combined with bovine-derived xenograft [57].

Several studies have examined the composition of grafts and their biological behavior. Mixing maxillary autogenous cortical bone with bovine- or porcine-derived xenograft in a 20:80 ratio demonstrated comparable biological, clinical, and radiological outcomes in terms of biomaterial resorption, osteoconduction, and osteogenesis [58].

Among the available substitutes, bovine-derived xenograft is one of the most widely used and extensively documented biomaterials for sinus lift surgery [59]. It exhibits strong osteoconductive properties but lacks osteoinductive capacity [60], and its slow or incomplete resorption may impair new bone formation [61]. By contrast, porcine-derived xenograft tends to resorb faster and promote new bone formation [18]. A recent in vivo study directly comparing bovine and porcine deproteinized grafts found no statistically significant differences in bone formation at 48 weeks [52]. However, when non-collagenated bovine and collagenated porcine xenografts were compared in sinus floor elevation, the collagenated xenograft underwent higher resorption, resulting in greater shrinkage of the elevated space but also a higher proportion of new bone in regions adjacent to the sinus walls [61].

THE Graft™ has a high degree of porosity, similar to human trabecular bone, which allows immediate absorption of biological fluids like blood [48]. Not only does this make it easy to handle and apply, but this property also facilitates early remodeling, which accounts for its excellent clinical performance.

### 4.4. Clinical Relevance and Future Perspectives

From a clinical perspective, porcine-derived xenografts represent a viable alternative to bovine-derived substitutes, as they avoid cultural or religious concerns while maintaining comparable regenerative capacity [62].

Recent studies support the clinical effectiveness of collagenated porcine xenografts in maxillary sinus augmentation, showing high rates of implant survival, favorable histologic maturation patterns, and stable volumetric results [58,63,64]. As clinical adoption continues to increase, new long-term comparative evidence further reinforces porcine xenografts as reliable and, in selected cases, potentially advantageous alternatives to bovine-derived grafting materials.

Furthermore, these materials exhibit good resorption behavior, which could lower the risk of late complications, such as chronic sinusitis, graft infection, persistent pain, or the formation of surgical ciliated cysts—features that might raise suspicions of poor long-term biocompatibility [26,65,66].

Consistent with previous reports, the presence of sinus pathology, such as an asymptomatic mucocele, did not compromise healing or implant survival [32,67].

The implant system used in the present cases was not considered a determining factor influencing the outcomes. The study aimed to document graft maturation rather than compare implant performance. Although implant macro- and micro-design may influence osseointegration, the implants were placed into fully healed augmented bone following a standardized protocol, and all cases showed uneventful integration. Given the small sample size, no conclusions can be drawn about implant-specific effects.

The present study contributes to the limited body of evidence by presenting paired clinical and histological outcomes, supported by long-term implant survival data. Excellent clinical handling, predictable regeneration, favorable histologic outcomes, and long-term implant function suggest that porcine xenografts are a safe and reliable option for the reconstruction of severely atrophic posterior maxillae. Larger controlled trials with extended follow-up are recommended to further validate these observations.

During this research, the patients shared satisfaction with the entire treatment process. They appreciated the opportunity to regain chewing function, aesthetics, and posterior support. They described a gradual recovery, without unexpected pain or complications. The patients expressed confidence in the procedure and in the final prosthetic outcome. This case series emphasizes that collagenated porcine xenografts can provide predictable bone regeneration and functional rehabilitation in severely atrophic maxillae.

Ongoing research in our clinic includes the collection and analysis of biopsy samples at nine months after grafting to further assess biomaterial resorption and new bone maturation over time.

Although the present study included only three cases, the findings remain relevant because human histological data after sinus augmentation are inherently limited. The collection of bone biopsies during implant osteotomy is not routine in most clinical settings due to cost, morbidity, and ethical constraints. Consequently, well-documented human histological samples are rare, and even small case series can provide valuable data on graft behavior and bone maturation.

## 5. Limitations of the Study

Nevertheless, this study has certain limitations. The small sample size, inherent to the case series design, restricts the validity of the results. Histological evaluation was based on trephine biopsies obtained at implant placement, which may not fully represent the entire grafted area. As this is a retrospective study from a single private clinic, potential selection bias cannot be excluded, and the outcomes may not be applicable to all clinical settings. Larger multi-center studies with extended follow-up and direct comparisons to alternative grafting materials are needed to validate and expand upon these preliminary findings.

## 6. Conclusions

- Within the limitations of this case series, small-particle (0.25–1.00 mm) collagenated porcine-derived xenograft demonstrated predictable clinical and radiographic performance in lateral sinus floor elevation.
- Stable long-term implant survival and significant vertical bone gain were achieved in the severely atrophic posterior maxilla.
- Histologic analysis confirmed the presence of vascularized new bone in intimate contact with residual graft particles, indicating favorable osteoconductivity and biocompatibility, with no adverse tissue reactions.
- These outcomes support the use of collagenated porcine xenograft as a safe and effective grafting option for maxillary sinus augmentation.

## Figures and Tables

**Table 2:** Clinical timeline according to CARE guidelines.

Time Point	Event	Key Details
Baseline (Month 0)	Pre-operative evaluation	Clinical exam; CBCT confirming severe posterior maxillary atrophy; treatment planning
Day 0	Lateral sinus augmentation	Collagenated porcine xenograft (THE Graft™); lateral window approach; one case: controlled Schneiderian membrane perforation for mucocele removal and collagen membrane repair
Weeks 1–2	Initial healing	No postoperative complications; uneventful soft tissue healing
Months 3–8	Implant placement and biopsy	Core specimens collected during implant osteotomy; histology (H&E, Masson–Goldner) + TRAP staining
3–4 months after implant placement	Prosthetic loading	Final implant-supported restorations delivered; functional loading commenced
Months 12–52	Follow-up period	Stable marginal bone levels (<1 mm first year); no complications; graft volume maintained
46.8 months (mean)	Long-term evaluation	All implants functional; radiographic and clinical stability confirmed
52.2 months (max)	Long-term	CBCT and periapical radiographs from available case demonstrating stable bone height and osseointegration

## Data Availability

The data presented in this study are available on request from the corresponding author.

## Abbreviations

The following abbreviations are used in this manuscript:

CBCT	cone-beam computed tomography
OPG	orthopantomogram
EDTA	ethylenediamine tetraacetic acid
HE	hematoxylin eosin
TRAP	tartrate-resistant acid phosphatase
BSM	xenogenic bone substitute material
NB	new trabecular bone
CT	connective tissue
MNGC	multinucleated giant cell
